# The neuromuscular effects of rocuronium under sevoflurane-remifentanil or propofol-remifentanil anesthesia: a randomized clinical comparative study in an Asian population

**DOI:** 10.1186/s12871-016-0231-0

**Published:** 2016-08-22

**Authors:** Sangseok Lee, Young Jin Ro, Won Uk Koh, Tomoki Nishiyama, Hong-seuk Yang

**Affiliations:** 1 Department of Anesthesiology and Pain Medicine, Sanggye Paik Hospital, In-Je University, College of Medicine, Seoul, South Korea; 2 Department of Anesthesiology and Pain Medicine, Seoul Asan Medical Center, University of Ulsan, College of Medicine, 88 Olympic-ro 43-gil, Songpa-gu, Seoul, 138-736 Korea; 3 Department of Anesthesiology, Shinagawa Shishokai Hospital, Tokyo, Japan

**Keywords:** Anesthetics inhalation, Sevoflurane, Anesthetics intravenous, Propofol, Remifentanil, Neuromuscular blocking agents, Rocuronium, Monitoring, Onset, Duration of action, Intubation condition

## Abstract

**Background:**

We conducted a prospective, randomized, multicenter study to evaluate the differences in the blocking effect of different doses of rocuronium between sevoflurane- or propofol-remifentanil anesthesia in an Asian population.

**Methods:**

A total of 368 ASA I–II patients was enrolled. Anesthesia was induced with 2.0 mg/kg propofol and 0.1 μg/kg/min remifentanil (TIVA) or 5.0 vol.% sevoflurane with 0.1 μg/kg/min remifentanil (SEVO). Tracheal intubation was facilitated at 180 s after the administration of rocuronium at 0.3, 0.6, or 0.9 mg/kg and then intubation condition was evaluated. The time to maximum block and recovery profile were monitored by TOF stimulation of the ulnar nerve and by recording the adductor pollicis response using acceleromyography.

**Results:**

The numbers of patients with clinically acceptable intubation conditions were 41, 82, and 97 % (TIVA) and 34, 85, and 90 % (SEVO) at each dose of rocuronium, respectively. There were no significant differences in the time to maximum block between groups at each rocuronium dose. There were significant differences in the recovery to a train-of-four ratio of 90 % between the groups: 42.7 (19.5), 74.8 (29.9), and 118.4 (35.1) min (TIVA) and 66.5 (39.3), 110.2 (43.5), and 144.4 (57.5) min (SEVO) at 0.3, 0.6, and 0.9 mg/kg, respectively (*P* < 0.001).

**Conclusions:**

There are no significant differences in intubation conditions between propofol-remifentanil and sevoflurane-remifentanil anesthesia at the same dose of rocuronium. The type of anesthetic does not significantly influence the time to maximum block by rocuronium. Rocuronium at a dose of 0.9 mg/kg should be used for better intubation conditions with both anesthesia regimens in an Asian population.

**Trial registration:**

UMIN-CTR Clinical Trial (http://www.umin.ac.jp/ctr/index.htm; UMIN#000007289; date of registration 14^th^ February 2012).

## Background

Rocuronium bromide is a monoquaternary, aminosteroid, non-depolarizing, muscle relaxant. Its main advantage compared with other currently available muscle relaxants is its rapid onset time and intermediate duration of action. The standard dose (2 × ED_95_, 0.6 mg/kg) of rocuronium is reported to provide clinically acceptable (good or excellent) intubation conditions in all patients at 90 s after administration, and is comparable to 1 mg/kg (3 × ED_95_) of succinylcholine [1]. Clinically, anesthesiologists in Korea and Japan have sometimes experienced unacceptable intubation conditions after administration of a standard dose of rocuronium. It is unknown whether the ethnicity of the patient can directly affect rocuronium potency but Dahaba et al. [2] have reported geographic regional differences in the rocuronium dose–response relation and time course of action. They have shown that the ED_95_ of rocuronium is significantly higher in Chinese patients than in American patients (475 ± 155 μg/kg vs 362 ± 149 μg/kg, respectively). Hence, the standard dose of rocuronium might not provide clinically acceptable intubation conditions in Asian patients. The first purpose of this study was thus to evaluate the intubation conditions at 180 s after administration of three different doses of rocuronium to investigate whether 0.6 mg/kg was sufficient in a population of Asian patients.

The potentiation of the neuromuscular blocking effects of muscle relaxants by inhalational anesthetics is well known [3–5]. Consequently, inhalational anesthetics can decrease the dose requirement of muscle relaxants and prolong both the duration of action and recovery from neuromuscular block. The dose-response curves of rocuronium under sevoflurane, isoflurane, or desflurane shifts to the left compared with those obtained during propofol anesthesia [4]. The interaction of inhalational anesthetics and muscle relaxants is a time-dependent phenomenon and the potentiation effect varies among inhalational agents [5]. Sevoflurane markedly potentiates muscle relaxants, shortening the onset time compared with other anesthetics. Such potentiation is not clear during induction and only becomes significant during prolonged anesthesia [6]. Thus, the second purpose of this study was to compare time to maximum block, and recovery profiles of rocuronium between sevoflurane-remifentanil and propofol-remifentanil anesthesia.

## Methods

### Patients

**Fig. 1:**
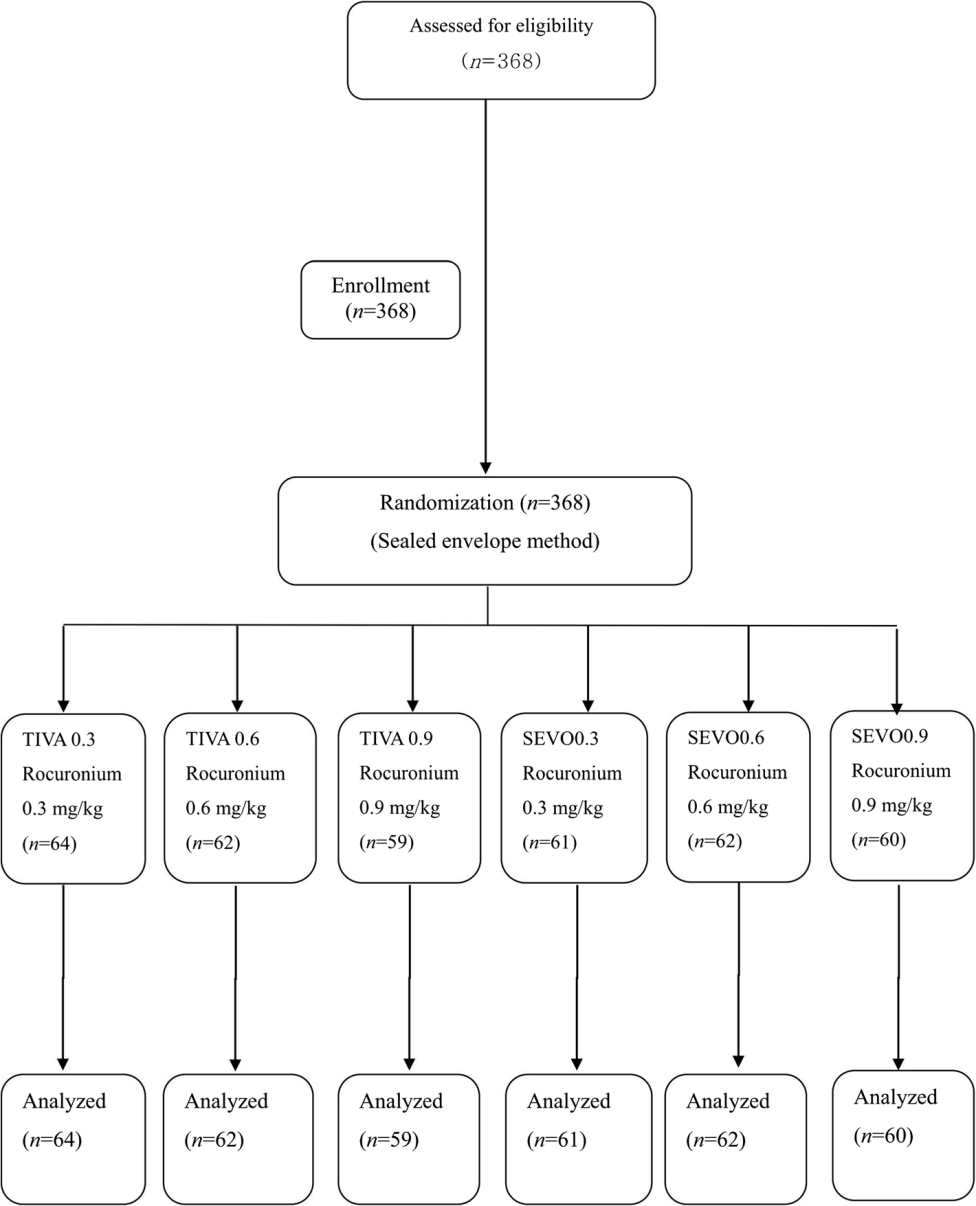
Flow diagram of the inclusion and exclusion criteria used in this study

This study was a prospective, multicenter, randomized, single-blind, parallel-group study performed in the South Korea and Japan (2 sites). After obtaining institutional review board approval (#016 at Higashi Omiya General Hospital and #2011-0697 at Asan Medical Center); registered with the UMIN clinical trial registry (www.umin.ac.jp), number UMIN#000007289 and written informed consent, we enrolled 368 Korean or Japanese ASA I or II patients, aged 20 to 80 years who would be undergoing elective general surgery with about 2 h of surgical duration (ex, laparoscopic cholecystectomy, laparoscopic appendectomy, simple mastectomy and etc.) under general anesthesia at Higashi Omiya General Hospital, Saitama, Japan and Seoul Asan Medical Center, Seoul, South Korea from February 2012 to February 2013. Using a sealed envelope method, 368 patients were randomly assigned to a sevoflurane (SEVO) or total intravenous anesthesia (TIVA) group. They were then randomly assigned to a rocuronium 0.3, 0.6, or 0.9 mg/kg subgroup (Fig. 1). Exclusion criteria were as follows: hepatic, renal, cardiac, respiratory, neurologic, or neuromuscular disease, hypertension, obesity (body mass index greater than 30), pregnancy, and drug allergies. The patients with Mallampati classification grade 3/4 or ones who were expected difficulties during intubation or laryngoscopic manipulation were excluded. In addition, any patients taking medications affecting neuromuscular blockade, such as anticonvulsants, anti-arrhythmic, and magnesium, were also excluded.

### Anesthesia technique and measurements

Anesthesia was induced with 2.0 mg/kg propofol and 0.1 μg/kg/min remifentanil (TIVA group) or 5.0 vol.% sevoflurane with 0.1 μg/kg/min remifentanil (SEVO group), and maintained with 4.0 mg/kg/h propofol and 0.1–0.5 μg/kg/min remifentanil (TIVA group) or 1.5–3.0 vol.% sevoflurane with 0.1–0.5 μg/kg/min remifentanil (SEVO group). Rocuronium was administrated when we confirmed the loss of consciousness after administration of propofol by checking the eyelash reflex and BIS under 40–50. If the patients would be still awake at 2 min after administration of propofol, additional propofol 0.5 mg/kg would be given. The supplement was not needed. Tracheal intubation using direct laryngoscopy with Macintosh blade without stylet was performed at 180 s after administration of rocuronium at 0.3, 0.6, or 0.9 mg/kg. The anesthesiologists who were blind to the subgroups, performed the intubation and assessed the intubating conditions and Cormack-Lehane (C-L) score. The intubation conditions as a primary endpoint were evaluated at 180 s after the administration of rocuronium using a modified grading system based on the system of Fuchs-Buder et al. [7] (Table 1). Systolic and diastolic blood pressure, heart rate, and bispectral index were measured before anesthesia and at 1, 3, and 5 min after intubation.

**Table 1:** Grade of intubating condition

Grade	Jaw relaxation	Vocal cord	Response to intubation
Excellent	Good	Immobile	None
Good	Good	Moving	Minimal diaphragmatic movement only
Poor	Good	Moving or actively closing	Coughing or bucking
Impossible	Poor	Closed	Intubation not possible

Modified from Fuchs-Buder et al.[7]

### Neuromuscular monitoring

Neuromuscular function was assessed using acceleromyography of the adductor pollicis muscle (TOF-Watch; Organon Ltd., Dublin, Ireland) according to the guidelines of Fuchs-Buder et al [7]. After loss of consciousness (BIS value < 60), neuromuscular monitoring began immediately with train-of-four (TOF) stimulation (0.2 ms duration, frequency 2 Hz, 2 s duration with supramaximal current, repeated every 15 s). As a secondary outcomes, the time to maximum block of rocuronium (sec), recovery index (T_25_ to T_75_, sec), and the time required for the TOF ratio to recover to 90 % (T_90_, sec) were measured.

### Statistical analysis

We analyzed intubation conditions, time to maximum block, recovery index, and time to T_90_ recovery using one-way analysis of variance (ANOVA) followed by post-hoc multiple comparison with Bonferroni’s correction. Blood pressure, heart rate, and bispectral index during anesthesia were analyzed using repeated measured ANOVA followed by within-/between-group multiple comparison as a post-hoc test. We expected that one grade of difference in the ease of intubation would make a clinically significant improvement in the intubation condition. We needed a total 300 patients to achieve 90 % power and 5 % significance level which can provide a significant difference between more than 3 groups. To allow for dropouts as 20 %, sample size was increased to 60 patients in each group. We analyzed the data using SAS/STAT® software version 8.2 (SAS Institute Inc., Cary, NC) and GraphPad Prism version 6.00 for Windows (GraphPad Software, La Jolla, CA). A *P*-value < 0.05 was considered statistically significant.

## Results

All data are expressed as mean (SD) or number of patient (%) or median (minimum-maximum). Patient characteristics were shown in Table 2. There was no significant difference in C-L score between groups (Table 3). The BIS values were significantly decreased after anesthesia induction compared with baseline (before anesthesia) (*P* < 0.001) and the SEVO groups showed significantly lower values than the TIVA groups (*P* < 0.001) [data not shown]. The hemodynamic values (systolic and diastolic blood pressure, heart rate) also significantly decreased at 1, 3, and 5 min after intubation compared with baseline (before anesthesia) in both groups (*P* < 0.001), while the systolic/diastolic blood pressure and heart rate of the SEVO groups were significantly lower than those of the TIVA groups at 3 and 5 min after intubation [data not shown]. However, concerning values at 1 min after intubation as anesthetic depth of the intubation condition, there was no significant difference in diastolic blood pressure, heart rate and BIS except systolic blood pressure between groups (*P* > 0.05, Table 3).

**Table 2:** Demographic data for the study participants

	TIVA03 (*N* = 64)	TIVA06 (*N* = 62)	TIVA09 (*N* = 59)	SEVO03 (*N* = 61)	SEVO06 (*N* = 62)	SEVO09 (*N* = 60)
Age (yrs)	51.3 (16.5)	54.8 (15.6)	61.9 (9.4)	54.6 (15.5)	53.2 (15.3)	58.6 (15.3)
Sex (M/F)	23 / 41	23 / 39	10 / 49	27 / 34	29 / 33	20 / 40
Height (cm)	160.5 (10.4)	160.9 (11.0)	155.6 (7.8)	161.8 (10.1)	161.6 (9.7)	159.8 (10.3)
Weight (kg)	64.4 (12.5)	68.2 (12.6)	62.2 (9.7)	64.5 (10.0)	65.2 (12.1)	62.2 (10.1)

Data are expressed as mean (SD) or number of patients

**Table 3:** Intubation conditions, airway classification and hemodynamic profile

	TIVA03 (*N* = 64)	TIVA06 (*N* = 62)	TIVA09 (*N* = 60)	SEVO03 (*N* = 61)	SEVO06 (*N* = 62)	SEVO09 (*N* = 60)	*P*-value
Excellent	2 (3)	18 (29)	41 (68)	5 (8)	25 (40)	43 (72)	<0.001*
Good	24 (38)	33 (53)	17 (28)	16 (26)	28 (45)	11 (18)	
Poor	12 (19)	8 (13)	1 (2)	22 (36)	7 (11)	5 (8)	
Impossible	26 (41)	3 (5)	1 (2)	18 (30)	2 (3)	1 (2)	
Acceptable	26 (41)	51 (82)	58 (97)	21 (34)	53 (85)	54 (90)	<0.001*
Unacceptable	38 (59)	11 (18)	2 (3)	40 (66)	9 (15)	6 (10)	
C-L score	1 (1–2)	1 (1–2)	1 (1–3)	1 (1–2)	1 (1–3)	1 (1–2)	>0.05^**^
SBP (mmHg)	115 ± 19	120 ± 21	116 ± 21	106 ± 20	110 ± 21	102 ± 19	>0.05^**^
DBP (mmHg)	70 ± 12	70 ± 13	68 ± 15	63 ± 14	66 ± 12	64 ± 13	>0.05^**^
HR (bpm)	72 ± 13	72 ± 14	74 ± 13	67 ± 13	68 ± 11	71 ± 13	>0.05^**^
BIS	44 ± 11	43 ± 14	41 ± 11	40 ± 13	42 ± 13	38 ± 11	>0.05^**^

All data are expressed as number of patients (%) or median (minimum-maximum). C-L score; Cormack-Lehane score for the grading of direct laryngoscopy. SBP; systolic blood pressure at 1 min after intubation. DBP; diastolic blood pressure at 1 min after intubation. HR; heart rate at 1 min after intubation. BIS; bispectral index at 1 min after intubation

* The difference within and between groups (TIVA and SEVO groups) at the same dose of rocuronium

There were no significant differences between the TIVA and SEVO groups at the same dose of rocuronium (*P* = 0.065 at 0.3 mg/kg, *P* = 0.612 at 0.6 mg/kg, and *P* = 0.262 at 0.9 mg/kg)

^**^ There was no significant difference in C-L grade between groups (*P* > 0.05). There was also no significant difference in SBP, DBP, HR and BIS at 1 min after intubation between groups (*P* > 0.05)

Intubating conditions were significantly different within and between groups (TIVA and SEVO groups) at the different doses of rocuronium (Table 3). However, there were no significant differences between the TIVA and SEVO groups at the same dose of rocuronium.

Larger doses of rocuronium significantly shortened the time to maximum block (Table 4). The differences between groups were not significant at the same dose of rocuronium. Larger doses of rocuronium significantly increased the recovery index in each group. The SEVO group showed a significantly longer recovery index than the TIVA group at the same dose of rocuronium. Larger doses of rocuronium showed a significantly longer recovery time to a TOF ratio of 90 % in both TIVA and SEVO groups. At the same dose of rocuronium, the SEVO group showed a longer recovery time to a TOF ratio of 90 % than the TIVA group.

**Table 4:** The time to maximum block and the recovery profile

	TIVA03 (*N* = 64)	TIVA06 (*N* = 62)	TIVA09 (*N* = 59)	SEVO03 (*N* = 61)	SEVO06 (*N* = 62)	SEVO09 (*N* = 60)	*P*-value
Maximum block (sec)	260.6 (130.1)	149.8 (62.5)	107.4 (43.3)	265.5 (143.0)	165.8 (91.8)	110.6 (38.8)	<0.001*
Recovery index (T_25_-T_75_) (min)	11.4 (4.4)	16.0 (4.6)	27.3 (8.5)	20.7 (16.5)	33.8 (20.6)	47.1 (20.7)	<0.001*^,^**
Recovery to T_90_ (min)	42.7 (19.5)	74.8 (29.9)	118.4 (35.1)	66.5 (39.3)	110.2 (43.5)	144.4 (57.5)	<0.001***

All data are expressed as mean (SD)

*Differences within groups (TIVA group or SEVO group)

**Differences between groups at the same dose of rocuronium

## Discussion

The results of our current study show that sevoflurane and propofol do not have different actions on the onset of the effects of rocuronium or on intubation conditions. However, sevoflurane significantly prolongs the duration of action of rocuronium compared with propofol. In addition, 0.9 mg/kg rocuronium was necessary to achieve adequate conditions for intubation under both propofol- and sevoflurane-remifentanil anesthesia in an Asian population.

Previously, Dahaba et al. [2] reported that there is a significant difference in rocuronium potency and duration of action among Austrian, Chinese, and American patients. According to their report, the ED_95_ of rocuronium in Chinese patients is 0.48 ± 1.6 mg/kg, which is significantly higher than the dose in American patients (0.36 ± 1.5 mg/kg). We show in our current investigation that clinically acceptable intubation conditions were achieved for 82 % (TIVA group) and 85 % (SEVO group) of patients after 0.6 mg/kg of rocuronium. However, for 0.9 mg/kg of rocuronium, 97 % (TIVA group) and 90 % (SEVO group) of patients showed acceptable intubation conditions (*P* < 0.001) and there was no difference between the types of anesthetic. Hence, we confirmed that the administration of 0.9 mg/kg of rocuronium can provide better intubation conditions under both propofol-remifentanil and sevoflurane-remifentanil anesthesia.

Several reports have shown that inhalational agents potentiate the neuromuscular effects of rocuronium. The mechanism by which inhalational anesthetics potentiate the effects of muscle relaxants is unknown. The proposed mechanisms include a central effect on alpha-motor neurons and interneuronal synapses [8], inhibition of postsynaptic nicotinic acetylcholine receptors [9], or augmentation of the antagonist’s affinity at the receptor site [10]. In addition, more than one mechanism is simultaneously involved and different inhalational anesthetics may not act exactly in the same way [11].

Such potentiation is not evident during induction and only becomes significant as the anesthesia duration becomes more prolonged [6]. In a study to quantify the relationship between the dose–response curve of vecuronium and the duration of exposure to an end-tidal concentration of sevoflurane, Suzuki et al. [5] showed that the duration of sevoflurane anesthesia influenced the dose–response of vecuronium and that 30 min inhalation of 1.7 % end-tidal concentration was sufficient to achieve a stable potentiating effect. In another report, 30 to 80 min was required to achieve maximal neuromuscular effects under 1 MAC halothane and isoflurane [12].

No report to date has shown that propofol could clinically potentiate neuromuscular blocking effects, but propofol has been reported to potentiate the effects of vecuronium, pancuronium, and suxamethonium in vitro [13]. Interestingly, intravenous anesthetics may have a direct effect on skeletal muscle [14]. Opioids are commonly used for anesthesia and are often administered with muscle relaxants. Opioids theoretically could affect neuromuscular blocking agents by reducing acetylcholine release [15]. However, we used remifentanil similarly in both our SEVO and TIVA groups and, therefore, the effects of remifentanil can be assumed the same in both groups.

We find from our current analyses that the type of anesthetic did not influence the time to maximum block. At the same dose of rocuronium, the mean time was similar between the TIVA and SEVO groups, but a higher dose of rocuronium shortened the time to maximum block. Furthermore, we found that there were no significant differences in the intubation conditions between the TIVA and SEVO groups at the same dose of rocuronium and that an increased rate of acceptable intubation conditions occurred with larger doses of rocuronium in both groups. These findings are in agreement with those reported by Lowry et al. [16], which showed that the onset time of mivacurium did not differ between sevoflurane and propofol. Ahmed et al. [17] also reported that an increase in sevoflurane exposure time did not shorten the time to maximum block. However, Yamaguchi et al. [18] reported that 8 % sevoflurane induction accelerates the onset of the vecuronium neuromuscular blockade. They found that the maximum block in the 8 % sevoflurane group was shorter than that in the propofol/fentanyl group and the N_2_O/2 % sevoflurane group (139 ± 35 s, 193 ± 35 s, and 188 ± 47 s, respectively). This finding is contrary to our present results. In their study, Yamaguchi et al. [18] administered vecuronium intravenously at 3 min after the start of anesthetic induction with sevoflurane, and the end-tidal concentration of sevoflurane reached between 6 and 7 % in the sevoflurane 8 % group. Tracheal intubation was performed approximately 2 min after administration of vecuronium. In our present study, we performed tracheal intubation and assessed the intubation conditions at 3 min after administration of rocuronium. Thus, the concentration of sevoflurane could be much less than that used by Yamaguchi et al. [18].

Cannon et al. [19] reported that patients receiving inhalational anesthetics require significantly lower vecuronium infusion rates to achieve a 90 % blockade than those receiving fentanyl, which represents a change in the pharmacodynamics of vecuronium-induced neuromuscular blockade rather than a change in the pharmacokinetics. The authors studied the effects of enflurane, isoflurane, and fentanyl, each in combination with 60 % nitrous oxide, on the vecuronium infusion rate necessary to maintain a constant 90 % depression of control muscle twitch tension. Yamaguchi et al. [18] also reported that the clinical duration from maximal block to 25 % recovery of the TOF ratio in two sevoflurane groups (2 and 8 %) was longer than that in a propofol/remifentanil group (47 ± 15, 48 ± 14, and 36 ± 10 min, respectively). Lowry et al. [16] studied the potency and time course of action of rocuronium in patients anesthetized with 66 % nitrous oxide in oxygen and 1.5 MAC sevoflurane, isoflurane, or propofol infusion. These authors reported that the mean ED_50_ and ED_95_ doses during sevoflurane anesthesia were significantly lower than those during propofol anesthesia. The recovery index and the times to recovery of T_l_ to 90 % and TOF ratio to 0.8 in the sevoflurane group were all significantly longer than in the propofol group. This accords well with our current results, where our SEVO groups showed a significantly longer recovery index than our TIVA groups at each dose of rocuronium. Furthermore, our SEVO groups showed a significantly longer recovery time to T_90_ than the TIVA groups. We therefore confirmed that the type of anesthetic could influence the recovery from neuromuscular block.

This study may have several limitations. First, our study results should be applied only to an Asian population. Second, we compared two anesthetic protocols with sevoflurane-remifentanil and propofol-remifentanil. Remifentanil infusion with sevoflurane in SEVO group rather than sevoflurane alone can be a confounding factor for interpretation of the intubation condition and the depth of anesthesia. Nevertheless, the reason we adopted this study design is that the protocol of this study was based on the daily practice protocol used in the clinical practice in Korea and Japan, and the results of of our study can be a real help to them. Third, we did not present BIS, hemodynamic variable, end-tidal sevoflurane concentration at the time of tracheal intubation. We considered the anesthetic depth by values measured at 1 min after intubation. There was no significant difference in diastolic blood pressure, heart rate and BIS except systolic blood pressure between groups. Therefore, we estimated that the same depth of anesthesia was provided to each group. However, some readers may not agree on that point. Lastly, sample sizes are approximate because calculation was based on our clinical assumptions not from previous references.

## Conclusions

In conclusion, we here demonstrate that to achieve a better and faster acceptable intubation conditions in Korean and Japanese patients, 0.9 mg/kg of rocuronium should be administered. However, sevoflurane-remifentanil anesthesia could prolong the recovery from neuromuscular block by rocuronium in comparison with propofol-remifentanil anesthesia.

## Abbreviations

ANOVA: Analysis of variance
ASA: American Society of Anesthesiology
BIS: Bispectral index
ED95: 95 % effective dose
SEVO: Sevoflurane
TIVA: Total intravenous anesthesia
TOF: Train-of-four
